# Icariin Promotes Tendon-Bone Healing during Repair of Rotator Cuff Tears: A Biomechanical and Histological Study

**DOI:** 10.3390/ijms17111780

**Published:** 2016-10-25

**Authors:** Chenyi Ye, Wei Zhang, Shengdong Wang, Shuai Jiang, Yuanbin Yu, Erman Chen, Deting Xue, Jianzhong Chen, Rongxin He

**Affiliations:** 1 Department of Orthopedic Surgery, the Second Affiliated Hospital, School of Medicine, Zhejiang University, No. 88, Jiefang Road, Hangzhou 310009, China; yechenyi@zju.edu.cn (C.Y.); zhangweilook@zju.edu.cn (W.Z.); 21518572@zju.edu.cn (S.W.); 3090103847@zju.edu.cn (S.J.); yybbyy@hotmail.com (Y.Y.); 21318096@zju.edu.cn (E.C.); blueskine@hotmail.com (D.X.); 2 Orthopedics Research Institute of Zhejiang University, No. 88, Jiefang Road, Hangzhou 310009, China; 3 Department of Hand Surgery, the First Affiliated Hospital, School of Medicine, Zhejiang University, No. 79 Qingchun Road, Hangzhou 310009, China; 4 Institute of Immunology, School of Basic Medical Sciences, Zhejiang University, No. 866, Yuhangtang Road, Hangzhou 310000, China; chenjianzhong@zju.edu.cn

**Keywords:** icariin, tendon-bone healing, rotator cuff tears

## Abstract

To investigate whether the systematic administration of icariin (ICA) promotes tendon-bone healing after rotator cuff reconstruction in vivo, a total of 64 male Sprague Dawley rats were used in a rotator cuff injury model and underwent rotator cuff reconstruction (bone tunnel suture fixation). Rats from the ICA group (*n* = 32) were gavage-fed daily with ICA at 0.125 mg/g, while rats in the control group (*n* = 32) received saline only. Micro-computed tomography, biomechanical tests, serum ELISA (calcium; Ca, alkaline phosphatase; AP, osteocalcin; OCN) and histological examinations (Safranin O and Fast Green staining, type I, II and III collagen (Col1, Col2, and Col3), CD31, and vascular endothelial growth factor (VEGF)) were analyzed two and four weeks after surgery. In the ICA group, the serum levels of AP and OCN were higher than in the control group. More Col1-, Col2-, CD31-, and VEGF-positive cells, together with a greater degree of osteogenesis, were detected in the ICA group compared with the control group. During mechanical testing, the ICA group showed a significantly higher ultimate failure load than the control group at both two and four weeks. Our results indicate that the systematic administration of ICA could promote angiogenesis and tendon-bone healing after rotator cuff reconstruction, with superior mechanical strength compared with the controls. Treatment for rotator cuff injury using systematically-administered ICA could be a promising strategy.

## 1. Introduction

Rotator cuff tears (RCT) are among the most common injuries seen by orthopedic surgery departments and can lead to recurrent pain and disability [1,2,3]. Rotator cuff (RC) reconstruction is currently the gold standard for the treatment of RCT. It has been estimated that over 270,000 RC reconstructions are performed in the United States each year, and the rate is continuing to increase [4,5,6]. Patients with an intact RC after reconstructive surgery tend to have notably improved functional outcomes [6,7,8]. However, despite significant advances in surgical techniques, particularly in terms of arthroscopic repair, RC repair remains linked to a high incidence of incomplete healing and re-tearing [9,10,11,12].

Clinical RCT in humans often occurs at the tendon-bone insertion site, which is composed of four typical tissue zones: tendon, fibrocartilage, mineralized fibrocartilage, and bone [13]. RC reconstruction creates a new tendon-bone interface along the bone tunnel. Because of bone loss at the RC reconstruction site and the avascularity of the fibrocartilage zone, the tendon-bone junction heals slowly and is the weakest point in the repaired RC, which subsequently increases the risk of failure after reconstructive surgery [2,7,8,14,15,16]. Recent strategies to promote osteogenesis, as well as fibrocartilage and mineralized fibrocartilage formation, at the tendon-bone insertion site have gained increasing interest within the field of orthopedic basic science [17,18].

Icariin (ICA, C_33_H_40_O_15_, molecular weight: 676.67) is the main active compound extracted from the well-established Chinese herb *Epimedium brevicornum* Maxim. ICA was originally proven to be protective against metabolic bone disease, especially osteoporosis [19,20,21]. Previous in vivo studies have shown that ICA could prevent ovariectomy-induced bone loss and restore femoral strength [20,21]. In postmenopausal women, bone mineral density (BMD) and bone strength were significantly improved after treatment by ICA [22,23]. In vitro studies have revealed that increased BMD after ICA treatment was associated with differentiation of bone marrow stromal cells (MSCs) into osteoblasts via estrogen receptor-mediated extracellular signal-regulated kinase (ERK) and c-Jun N-terminal kinase (JNK) signal activation, as well as with the enhanced expression of various proteins critical to bone matrix deposition, including osteoprotegerin (OPG), bone morphogenetic protein (BMP), etc. [24,25,26,27]. Osteoclast formation was also significantly inhibited after the application of ICA [28]. In addition, it has been reported that ICA was able to reverse the phenotype of OPG-deficient mice through activation of the Wnt/β-catenin/BMP signaling pathways [29]. Notably, several studies have also shown that ICA can promote angiogenesis [30]. An in vivo study of a mouse calvarial defect model by Zhao et al. showed that ICA significantly improved blood vessel formation, together with the formation of new bone [30].

A growing number of studies have shown that factors that promote osteogenesis and inhibit bone resorption, such as osteoinductive growth factors [31,32,33], also positively affect tendon-bone healing [34]. Treatments that enhance angiogenesis can also positively accelerate tendon repair [34,35,36], improve osteogenesis [17,18,37,38] and chondrogenesis [39], and theoretically promote tendon-bone healing [34,40,41,42]. However, the effects of ICA on tendon-bone healing remain unclear. The objective of this study was to evaluate the effect of ICA on RCT healing through structural, biomechanical, radiographic, and histological assessment in a rat model [43].

## 2. Results

### 2.1. Micro-CT Analysis Found ICA Enhanced New Bone Formation and Bone Quality

**Figure 1:**
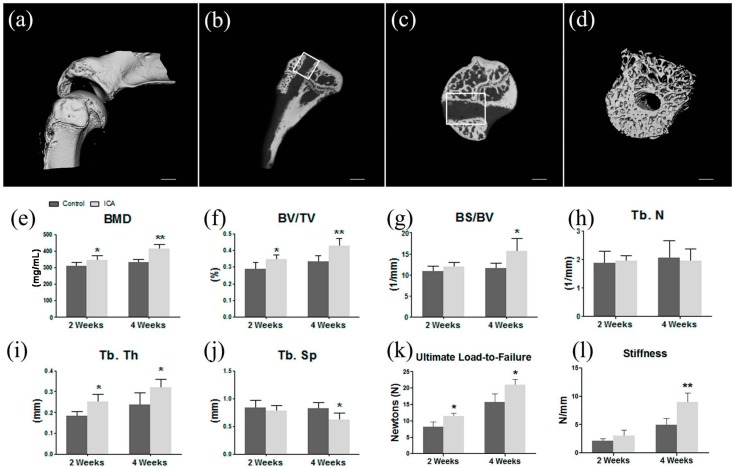
A sketch map and results of the micro-CT evaluations and biomechanical testing: (**a**) Gross observation of specimen containing the supraspinatus and humerus of the surgical side in a three-dimensional reconstruction micro-computed tomography (micro-CT) image; (**b**) The vertical plane of the axis of the humerus bone tunnel in a sagittal view on the micro-CT image; new bone formation in the region of interest (ROI) within the bone tunnel, and the bone density at the tendon insertion site on the greater tuberosity, was revealed and evaluated (white rectangle); (**c**) Cross section of the axis of the humerus bone tunnel on the micro-CT image; the ROI is shown with new bone within the bone tunnel (white rectangle); (**d**) The cylinder scope including the bone tunnel with a diameter of 1.5 mm and depth of 3.0 mm from the joint surface in a three-dimensional reconstruction micro-CT image; (**e**–**j**) The results of micro-CT analysis revealed that ICA treatment effectively improved the bone quality. BS/BV: bone surface/volume ratio; BV/TV: bone volume fraction; Tb.Th: trabecular thickness; Tb.Sp: trabecular separation; Tb.N: trabecular number; (**k**,**l**) At two weeks, the mean ultimate load-to-failure was significantly higher in the ICA group compared with the control group. There was no significant difference in stiffness at two weeks between the two groups. At four weeks after operation, the average failure load and mean stiffness in the ICA group were significantly greater than in the control group. Scare bar = 1 mm. * *p* < 0.05 vs. the control group; ** *p* < 0.01 vs. the control group.

The results of the micro-CT analysis revealed that the averages of the BMD in the ICA group were significantly higher than those in the control group (two weeks: ICA, 0.3472 ± 0.0273 mg/mL vs. control, 0.3122 ± 0.0138 mg/mL; *p* = 0.044, *n* = 8; four weeks: ICA, 0.4437 ± 0.0264 mg/mL vs. control, 0.3301 ± 0.0181 mg/mL; *p* = 0.001, *n* = 8). Compared to the controls, ICA application significantly increased the BS/BV, BV/TV, and Tb.Th of the area selected at four weeks postoperatively. In addition, ICA significantly decreased the Tb.Sp at four weeks postoperatively. No significant difference in Tb.N was detected between the two groups (Figure 1).

### 2.2. Biomechanical Testing Showed that ICA Increased the Bonding Strength at the Tendon-Bone Interface

In the evaluation of the failure load, all specimens failed by total pullout from the bone tunnels and no specimens were discarded. At two weeks, the mean ultimate load to failure was significantly higher in the ICA group compared with the control group (11.5 ± 2.0 N vs. 8.2 ± 1.5 N, respectively; *p* = 0.016; *n* = 8). There was no significant difference in stiffness at two weeks between the two groups (ICA, 3.1 ± 1.0 N/mm vs. control, 2.1 ± 0.4 N/mm; *p* = 0.083, *n* = 8).

At four weeks after operation, the average of the failure load in the ICA group was significantly greater than that in the control group (ICA, 21.0 ± 3.7 N vs. control, 15.8 ± 2.5 N; *p* = 0.031, *n* = 8). Additionally, the mean stiffness was significantly higher in the ICA group compared with the control group at four weeks postoperatively (9.0 ± 1.6 N vs. 5.0 ± 1.1 N, respectively; *p* = 0.002; *n* = 8) (Figure 1).

Compared to the healthy contralateral side, all groups had a significant lower load to failure and stiffness. No significant difference regarding the load to failure ratio (treated/contralateral) or stiffness ratio was found between control group and ICA treated group in two weeks after operation. However, the results of our study showed ICA treatment significantly improved the load to failure ratio in four weeks postoperatively, while the improvement of ICA on stiffness ratio did not reach significance (Figure S1).

### 2.3. Serum ELISA Revealed that ICA Increased the Serum Level of Osteogenic Markers, Including Ca, AP, and OCN

**Figure 2:**
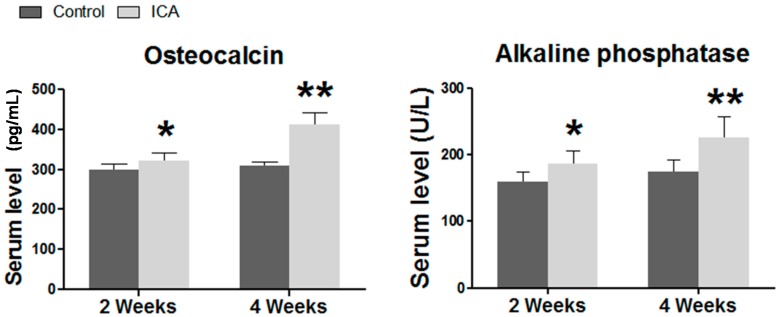
Serum chemistry. The results of ELISA showed that the application of ICA significantly enhanced osteogenesis. The expression of alkaline phosphatase (AP), and osteocalcin (OCN) in the ICA group were significantly increased at two and four weeks postoperatively. * *p* < 0.05 vs. the control group; ** *p* < 0.01 vs. the control group.

The results of ELISA showed that the application of ICA significantly enhanced the protein expression of AP and OCN at two weeks after surgery (*p* < 0.05). At four weeks after surgery, the serum levels of AP, and OCN were significantly increased in the ICA group when compared with the samples in the control group (*p* < 0.05) (Figure 2). Interestingly, a trend towards higher serum calcium level was observed in the ICA treated group (Figure S2).

### 2.4. Immunohistochemical Analysis Showed that ICA Promoted Angiogenesis and Accelerated Tendon-Bone Healing

**Figure 3:**
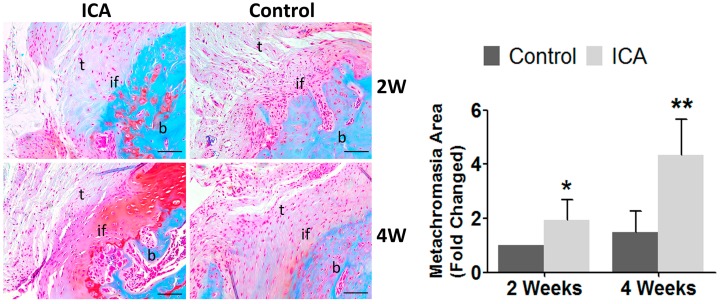
Representative images (total magnification, 100×) of Safranin O/Fast Green staining to assess fibrocartilage formation at the enthesis. Stronger proteoglycan staining was observed at the tendon-bone interface in the ICA group compared to the control group. Larger area of cartilage present at the insertion site as determined by metachromasia with safranin O–stained were observed in the ICA group, when compared with the control group. 2W: two weeks postoperatively; 4W: four weeks postoperatively; t: tendon; b: bone; if: tendon-bone interface. Semi-quantitative analyses were conducted using Image J software (National Institutes of Health). * *p* < 0.05, ** *p* < 0.01, bar = 200 μm.

The results of Safranin O and Fast Green staining showed that there were significantly increased area of metachromasia in the ICA treated group than the control groups at both two and four weeks after surgery. In addition, larger collection of cartilage cells at the tendon-bone interface was observed in the ICA group than in the control group (Figure 3).

**Figure 4:**
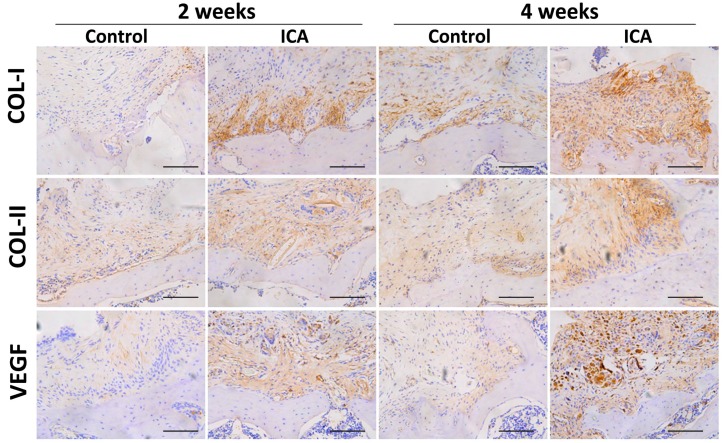
Results of the immunohistochemical staining. ICA treatment significantly increased the expression of type I and type II collagen when compared with the level in the control group at each time point (total magnification, 200×). In addition, more VEGF-positive cells were observed in the ICA group, revealing that ICA may not only improve the formation of collagen, but could also improve angiogenesis, bar = 100 μm.

The results of immunohistochemical staining showed a significant increase in the expression of Col1 and Col2 in the ICA group compared with the level in the control group at each time point. Notably, more VEGF-positive cells were observed in the ICA group compared with the control group (Figure 4).

**Figure 5:**
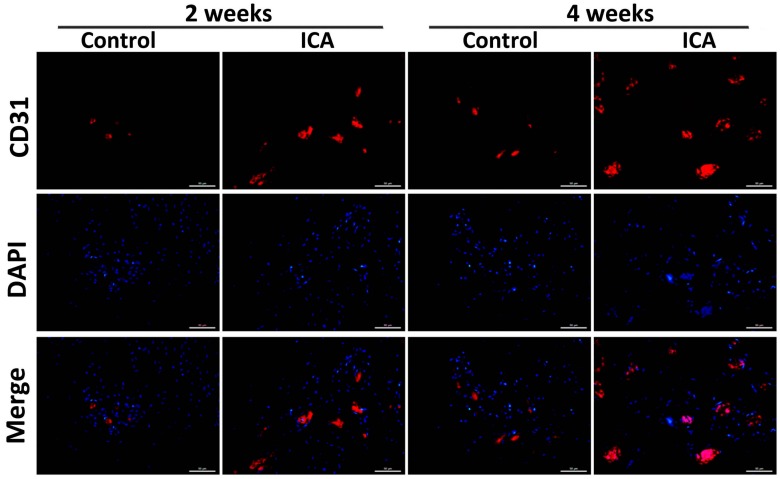
Results of the immunofluorescence staining of CD31. The results of immunofluorescence staining of the samples showed that the CD31 protein expression was significantly increased after ICA treatment, bar = 50 μm.

**Figure 6:**
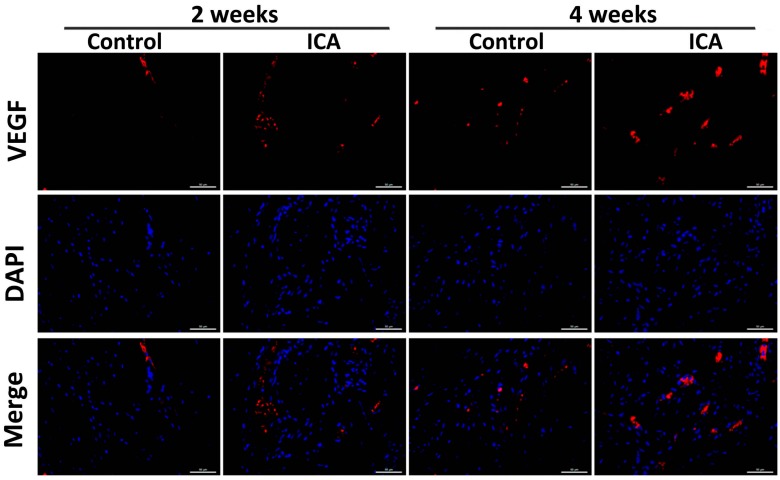
Results of the immunofluorescence staining of VEGF. The results of immuno-fluorescence staining of the samples showed that the VEGF protein expression was significantly increased after ICA treatment, which confirmed the findings of immunohistochemical staining and demonstrated that ICA improved angiogenesis, bar = 50 μm.

Angiogenesis at the tendon-bone interface was also detected by immunefluorescence staining of endothelial markers. At both two and four weeks after surgery, vascular staining with CD31 and VEGF demonstrated an enhancement of intrinsic neovascularization around the tendon insertion site in the ICA group compared with that in the control group (Figure 4, Figure 5 and Figure 6), revealing that ICA increased both angiogenesis and osteogenesis.

In addition, the results of immunohistochemical and immunofluorescence staining showed that there was no significant difference regarding the Col3 protein expression between ICA treated group and the control group. These results were confirmed by semi-quantitative analyses using Image J software (National Institutes of Health) (Figure S3).

## 3. Discussion

To our knowledge, this is the first study to investigate the effects of ICA on tendon-bone healing. In the current study, the hypothesis that ICA, which has been shown to contribute to angiogenesis and osteogenesis, might contribute to tendon-bone healing and regeneration was proven. Using a rat RC reconstruction model, ICA was shown to have a therapeutic potential for early tendon-bone healing during the repair of RCT, confirmed by micro-CT, immunohistochemistry, histology, and biomechanical testing. Our findings were similar to previous studies, in that factors that promote osteogenesis and angiogenesis or inhibit bone resorption were reported to have a positive effect in terms of promoting tendon-bone healing [31,32,33]. Ma et al. used rhBMP-2, a powerful osteoinductive agent, in a rabbit anterior cruciate ligament (ACL) reconstruction model and demonstrated that rhBMP-2 treatment led to significantly increased stiffness [31]. In a controlled laboratory study of ACL reconstruction, Sasaki et al. [33] applied granulocyte colony-stimulating factor (G-CSF), which has been proven to contribute to angiogenesis [44,45], in 28 beagle dogs and demonstrated that the application of G-CSF significantly accelerates bone-tendon healing via enhanced angiogenesis and osteogenesis [33].

Osteogenesis, especially new bone formation and bone remodeling around the tendon, is the key step in the tendon-bone healing process [31,32,33,46]. In the current study, a greater degree of new bone formation towards the tendon in the tendon-bone interface was observed in the ICA-treated group. In addition, the results of this study showed that ICA application significantly increased the serum level of AP, and OCN proteins. The bone quality in the ICA-treated rats was also enhanced, which was consistent with previous studies [20,21,22,23,26,47]. These results suggest that the protective effects of ICA on tendon-bone healing during the repair of RCT may be due in part to its promoting effect on osteogenesis. Wang et al. conducted an in vivo study using a murine calvarial osteolysis model and demonstrated that ICA significantly promoted bone formation in a wear-debris-induced osteolytic site [47]. The in vitro research of their study showed that ICA significantly induced osteogenic differentiation of MSCs via activation of the Wnt/β-catenin signaling pathway [47]. In another in vitro study, Song et al. reported that ICA significantly promoted MC3T3-E1 osteoblastic cell proliferation and reduced cell apoptosis. ERK and JNK were also significantly activated [26]. The results of these studies provide a potential mechanism for the osteogenic actions of ICA involving the Wnt/β-catenin, ERK, and JNK signaling pathways. Further studies are, therefore, needed.

Angiogenesis is one of the most fundamental factors needed for tendon-bone healing [17,18,37,38,48,49]. An adequate blood supply is essential to transport nutrients, minerals, and oxygen for bone matrix synthesis and mineralization. There is increasing evidence that osteogenesis, angiogenesis, and tendonogenesis share common mechanisms involved in the differentiation of bone-marrow-derived MSCs [37,38,50]. In patients with RCT, the blood supply at the tendon insertion site is disrupted; this is considered to be one of the most important causes of incomplete healing and re-tearing [9,10,11,12]. In addition, neovascularization is essential for tendon remodeling at the tendon-bone junction [49,51]. Several previous studies have shown that angiogenesis is essential for tendon-bone healing by detecting CD31 and VEGF expression in the tendon-bone junction (19–21) [17]. ICA has also been shown to promote angiogenesis [52,53,54,55]. Chung et al. investigated the molecular effect of ICA on angiogenesis and demonstrated that ICA stimulates angiogenesis by activating the MEK/ERK and PI3K/Akt/eNOS-dependent signal pathways in human endothelial cells [52]. Xin et al. studied the effect of ICA on diabetic retinopathy in a rat model and found a significantly increased expression of VEGF in the retinal vessels of diabetic rats after treatment by ICA [55]. In 2015, Le et al. conducted an in vitro study and demonstrated that the endometrium may be thickened by ICA treatment, by increasing the expression levels of VEGF in endometrial cells [53]. Similarly, in our study, the histological results showed that ICA clearly enhanced angiogenesis, compared with the control group. Expression of the VEGF and CD31 proteins was significantly improved after ICA treatment. The regenerated tendon was significantly more regularly aligned in the ICA group than in the control group. Taking these findings together, we consider ICA-induced enhancement of angiogenesis to represent an important part of the protective effect of ICA on tendon-bone healing after RC reconstruction.

Col1 and Col2 are the predominant components of the tendon and fibrocartilage zone and are widely accepted as markers for tendon-bone healing [9,10,11,12]. The newly formed scar, however, is mainly composed of type III collagen, instead of Col1 and Col2. Galatz et al. [2] and Wei et al. [56] have demonstrated that the expression of Col1 and Col2 is an important indicator of the total repair outcome of RCT, especially at later stages during repair maturation. In the current study, a significant increase in Col1 and Col2 in the ICA group compared with those in the control group was observed at each time point, while no significant difference was found between ICA and control groups regarding the Col3 protein expression, which indicated the scar tissues cannot be decreased due to the treatment of ICA in early stage (2–4 weeks). Thus, the long-term follow-up is required to reveal whether or not the use of ICA contribute to scarless healing. However, it is encouraging that higher levels of Col1 and Col2 were observed. The results at four weeks postoperatively showed that specimens in the ICA group had significantly more fibrocartilage cells than those in the control group. This result suggested that the application of ICA may help promote the formation of fibrocartilage and enhance tendon-bone healing. The most plausible mechanism, we believe, may be due to the promoted angiogenesis by ICA, as outlined above. Further studies are, however, required.

ICA has been used in the treatment of bone fractures and osteoporosis in traditional Chinese medicine for centuries and few major side effects have yet been reported [47,57]. Qin et al. studied the influence of ICA on cell proliferation with serial concentrations of ICA (20, 40, 80, 160 or 320 μg/L) and found no side effect on cell growth [58]. Zeng et al. studied the effect of ICA on HumanSW1353 chondrosarcoma cells and found that treatment of cells with 5, 10, or 20 μM ICA did not affect cell viability [59]. In addition, negative results were demonstrated from genotoxicity experiments including mice bone marrow micro-nuclear test, Ames test and TK gene test. The Lethal dose, 50 percent kill (LD_50_) for rodent-mouse is reported to be 80 gm/kg [60]. Li et al. applied ICA at a dosage of 40 mg/kg in a murine osteoporotic model and found no adverse effect in the body and uterine weight of ovariectomized mice [61]. The safety of ICA was also confirmed by other in vivo studies [47,58,62]. Moreover, ICA has been used safely for up to eight weeks in one clinical trial [63]. Xiao et al. investigated the safety and efficacy of ICA in persons with a psychiatric illness at a dose up to 300 mg/day for eight weeks and observed no significant no side-effects [63]. Although no major side effects have yet been reported, ICA is considered about 1/80th as powerful of a PDE5 inhibitor, which may theoretically has a stimulatory effect and may affect vision, hearing and the stomach in very high doses [64,65,66]. More long-term toxicity studies about ICA are needed.

Some limitations of our study should be noted. First, this was an in vivo study that lacked an in vitro evaluation of the potential impact of ICA. Thus, the mechanism of angiogenesis enhancement by ICA was not studied, which may decrease the robustness of our results; Second, we evaluated the effects of ICA on the early stage of tendon-bone healing during repair of RCT (at two and four weeks), but the longer-term effects of ICA were not evaluated; Third, we used a well-established RC reconstruction model, as described previously [43,67,68,69,70], which differs from that used in humans. The single load-to-failure test construct in this study might not be identical to cases of RCT; Fourth, although we evaluated the effect of ICA on osteogenic markers, including AP, and OCN, the role of ICA therapy on calcium homeostasis and its mechanism, however, is not studied. In addition, it is possible that the improved load-to-failure may due to the ICA-induced new bone formation at the tendon-bone interface. However, the results of the current study are encouraging. Despite these limitations, this study provides useful insight into the potential effects of ICA on tendon-bone healing after RC repair. Future studies with better designed large-animal models and a longer timescale are warranted to examine the effectiveness of ICA for RC repair in humans.

## 4. Materials and Methods

### 4.1. Study Design

**Figure 7:**
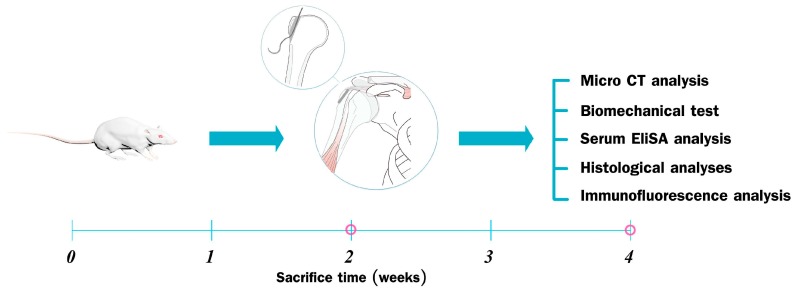
Study design in vivo. All Sprague Dawley rats were randomly divided into a control group and an experimental (icariin; ICA) group (*n* = 32 per group). Once the rat rotator cuff tear model was established using bone tunnel suture fixation, rats in the ICA group were gavage-fed daily with ICA at 0.125 mg/g/day, while animals in the control group received the same volume of saline. The rats were sacrificed at weeks two and four, and specimens containing the supraspinatus and humerus of the surgical side were collected for subsequent analysis.

The study protocol was approved by the Institutional Animal Care and Use Committee of the Second Affiliated Hospital, School of Medicine, Zhejiang University (Register ID No.: 2015-024; Date: 5 February 2015), strictly following the guidelines for the care and use of laboratory animals. All animals were supplied by the Academy of Medical Sciences of Zhejiang Province. The animals had free access to food and water and were kept in a pathogen-free animal room. In total, 64 male Sprague Dawley rats (weight, 250–300 g) were used to establish a rat RCT model, as described previously [67,68,69,70]. The rats were divided randomly and evenly into two groups: a control group and an experimental (ICA) group (*n* = 32 per group). All animals underwent the complete detachment and immediate repair of the right supraspinatus tendon with bone tunnel suture fixation, as described previously [67,68,69,70,71]. Once the rat RCT model was established, animals in the ICA group were gavage-fed daily with ICA at 0.125 mg/g [20,21,62], while animals in the control group received the same volume of saline. The animals were then sacrificed in a CO_2_ chamber at two or four weeks postoperatively (*n* = 16 in each group at each time point). Specimens containing the supraspinatus and humerus of the surgical side were collected for biomechanical testing, radiographic analysis, and histological analysis (*n* = 6 in each group for each analysis at each time point). All authors were blinded to the study groups and time intervals of the specimens at the time of the histological analysis and biomechanical testing (Figure 7).

### 4.2. Surgical Procedure

All surgical procedures were performed under strictly aseptic conditions using a previously described protocol [67,68,69,70,71]. The rats were anesthetized with an intraperitoneal injection of pentobarbital sodium solution (Kyoritsu Seiyaku, Tokyo, Japan; 50 mg/kg body weight). The rat was placed in the lateral decubitus position. After carefully shaving the hair overlying the operation area and administering topical sterilization three times, a 1-cm longitudinal incision using an open deltoid-splitting approach was made on the anterolateral aspect of the shoulder to identify the RC musculature and the insertion of the supraspinatus tendon on the greater tuberosity of the proximal humerus. A modified Mason-Allen stitch was then applied using 5-0 Prolene sutures (Ethicon, Blue Ash, OH, USA) to mark the supraspinatus tendon. The tendon was then detached sharply from the greater tuberosity, and the existing adhesions were released from the footprint. Using a 0.5-mm diameter Kirschner wire (Zimmer, Warsaw, IN, USA), a bone tunnel from the anteriomedialis to posterolateral of the greater tuberosity was created at the insertion site of the supraspinatus tendon. The free suture ends of the supraspinatus tendon were then passed through the bone tunnels and tied over the humeral cortex to ensure that more of the supraspinatus tendon was fixed in the bone tunnels. The wound was then closed in a standard layered fashion. All rats were then returned to their cages and allowed to move freely. Weight-adjusted pain medication (buprenorphine, 0.05 mg/kg) was administered subcutaneously every 12 h for analgesia for a period of three postoperative days [67,68,69,70,71].

### 4.3. Serum Chemistry

Blood samples were collected from the aorta abdominalis (*n* = 8/group, volume = 4 mL for each rat) at the time of sacrifice. Serum was separated immediately using centrifugation at a speed of 3000 r/min for 10 min and was collected and stored at −80 °C for ELISA analysis. The serum levels of alkaline phosphatase (AP) and calcium (Ca) were tested using an automatic biochemical analyzer (Au5400, Beckman Coulter Inc., Massachusetts, CA, USA), while the serum level of osteocalcin (OCN) was analyzed using a specific kit (Rapidbio Inc., Los Angeles, CA, USA).

### 4.4. Biomechanical Testing

Biomechanical testing was performed at two and four weeks postoperatively (*n* = 8 for each group). All soft tissue, except for the humerus with the attached supraspinatus tendon, was carefully dissected to create a humerus-supraspinatus complex. The specimens were immediately frozen at −80 °C and thawed overnight at 4 °C for testing. All specimens were kept moist with normal saline (NS). The biomechanical test was performed using an Instron 553 A material testing system (Instron, Boston, MA, USA). Each supraspinatus tendon was fixed using a screw grip with sandpaper and each humerus was fixed in the other vice grip of the testing system.

**Figure 8:**
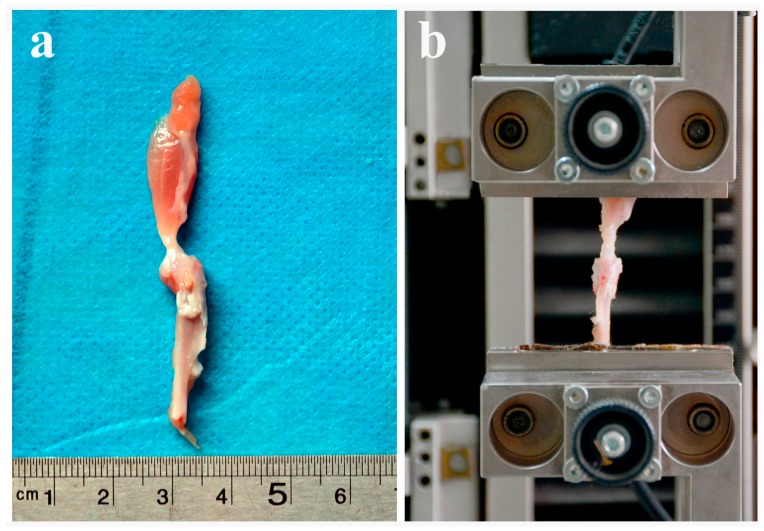
A sketch map of the biomechanical tests. The specimen containing the supraspinatus and humerus of the surgical side (**a**) was fixed to an Instron 553 A material testing system (Instron, Boston, MA, USA) to perform the biomechanical tests (**b**).

The grip-to-grip distance was standardized across all humerus–supraspinatus complexes. Each specimen was then preloaded to 0.10 N and the tensile load was then increased to failure with a crosshead speed of 5 mm/min. The maximum failure load (N) was recorded, and the stiffness (N/mm) was calculated from the load-deformation curves using Sigma Plot 8.0 (SPSS Inc., Chicago, IL, USA) (Figure 8).

In addition, biomechanical testing of the healthy contralateral side was also conducted using the same method of the operated side. The load to failure ratio (treated/contralateral) and stiffness ratio were calculated in order to assess the effectiveness of the therapy with respect to improvement of the return of the pullout strength of the tendon graft.

### 4.5. Micro-CT Analysis

The right shoulder joints (*n* = 8 for each group) were collected at two and four weeks postoperatively and fixed in 4% paraformaldehyde. Each shoulder joint sample included only the proximal third of the humerus and the supraspinatus tendon-bone complex. Micro-computed tomography (micro-CT; 36-μm thickness; 80 kV, 450 mA) (Skyscan1176; Bruker Corp., Antwerp, Belgium) was used to determine new bone formation in the bone tunnels and the bone density at the tendon insertion site on the greater tuberosity. Image-Pro Plus 6.0 software (IPP 6.0, Media Cybernetics Inc., Rockville, MD, USA) was used to measure each area three times. The BMD, bone surface/volume ratio (BS/BV), bone volume fraction (BV/TV), trabecular number (Tb.N), trabecular separation (Tb.Sp), and trabecular thickness (Tb.Th) were calculated for a volume of interest at the greater tuberosity, including the bone tunnel with a diameter of 1.5 mm and depth of 3.0 mm from the joint surface [67,72] (Figure 1).

### 4.6. Histomorphometry, Immunofluorescence, and Immunocytochemistry

After micro-CT scanning, the specimens (*n* = 8 for each group) were decalcified in 10% ethylene diaminetetra acetic acid with 0.1 M phosphate-buffered saline for 60 days, and then embedded in paraffin using standard procedures. Cross sections (3 μm) were cut in the coronal plane through the supraspinatus tendon insertion site on the greater tuberosity, pasted onto glass slides, and deparaffinized. Sections were stained with hematoxylin and eosin for traditional light microscopy (Leica DM4000B; Leica, Solms, Germany). Safranin O/Fast Green staining was also performed to allow the observation of fibrocartilage formation at the tendon-bone interface of the tendon insertion site [67,68,69,70,71].

For immunohistological staining of type I collagen (1:200) (Col1; Abcam, Shanghai, China), type II collagen (1:200) (Col2; Abcam), type III collagen (1:200) (Col3; Affinity) and vascular endothelial growth factor (1:200) (VEGF; Abcam), sections were incubated with primary antibodies overnight at 4 °C. Sections were then incubated with appropriate secondary antibodies for 30 min after washing, and thereafter rinsed and incubated with avidin-biotin enzyme reagent for 30 min at 37 °C. Finally, sections were counterstained with 3.3′-diaminobenzidine tetrahydrochloride and hematoxylin.

Immunofluorescence analysis of the sections containing bone and tendon was performed as described previously [73,74]. Briefly, sections with individual primary antibodies to rat VEGF (1:400; Santa Cruz Biotechnology, Shanghai, China), rat Col3 (1:200; Affinity Biosciences, Cincinnati, OH, USA) and rat CD31 (1:200; Santa Cruz Biotechnology) were incubated overnight at 4 °C. Secondary antibodies conjugated with a source of fluorescence at room temperature were subsequently applied for 2 h in the absence of light. Under the same conditions, isotype-matched antibodies (Santa Cruz Biotechnology) were used as negative controls. The number of positively-stained cells around the tendon insertion site on the greater tuberosity was determined in four random visual fields, in five sequential sections per rat in each group (*n* = 8), using a fluorescence microscope (EU5888; Leica, Wetzlar, Germany).

Semi-quantitative analyses of Safranin O/Fast Green staining, immunohistological and immunofluorescence analysis were performed using computerized image analysis (Image J, National Institutes of Health). Specific details of the methods for both of these analyses have been previously described [2].

### 4.7. Statistical Analysis

Based on previously reported studies [13,75,76], a priori power analysis with an error probability of 0.05 and a power of 0.80 revealed that a minimum sample size of eight rats per group per time point was necessary for biomechanical testing. Data are expressed as means ± standard deviation. Statistical analysis was performed using a one-way analysis of variance followed by Bonferroni’s post hoc test. A *p*-value of less than 0.05 was considered statistically significant.

## 5. Conclusions

This study suggests that the systematic administration of ICA promotes tendon-bone healing at an early stage. The results of this study may enable a new strategy for the promotion of tendon-bone healing after RC reconstruction. Further studies are, however, required.

## Supplementary Materials

Supplementary materials can be found at www.mdpi.com/1422-0067/17/11/1780/s1.

## Abbreviations

ICA	Icariin
SD	Sprague Dawley
Ca	Calcium
AP	Alkaline Phosphatase
OCN	Osteocalcin
Col	Collagen
VEGF	Vascular Endothelial Growth Factor
RCT	Rotator Cuff Tears
RC	Rotator Cuff
BMD	Bone Mineral Density
MSCs	Bone Marrow Stromal Cells
ERK	Estrogen Receptor-Mediated Extracellular Signal-Regulated Kinase
JNK	c-Jun N-terminal Kinase
OPG	Osteoprotegerin
G-CSF	Granulocyte Colony-Stimulating Factor
ACL	Anterior Cruciate Ligament
NS	Normal Saline
BS	Bone Surface
BV	Bone Volume
TV	Total Volume
Tb.N	Trabecular Number
Tb.Sp	Trabecular Separation
Tb.Th	Trabecular Thickness
