# Supplementary material for: Icariin Promotes Tendon-Bone Healing during Repair of Rotator Cuff Tears: A Biomechanical and Histological Study
Source: Int J Mol Sci. 2016 Oct 25;17(11):1780. doi: 10.3390/ijms17111780 (PMC5133781; doi:10.3390/ijms17111780)
Supplement: Supplementary file 1 [file ijms-17-01780-s001.pdf]

Supplementary Materials: Icariin Promotes Tendon-Bone Healing during Repair of Rotator Cuff Tears: A Biomechanical and Histological Study

Chenyi Ye, Wei Zhang, Shengdong Wang, Shuai Jiang, Yuanbin Yu, Erman Chen, Deting Xue, Jianzhong Chen and Rongxin He

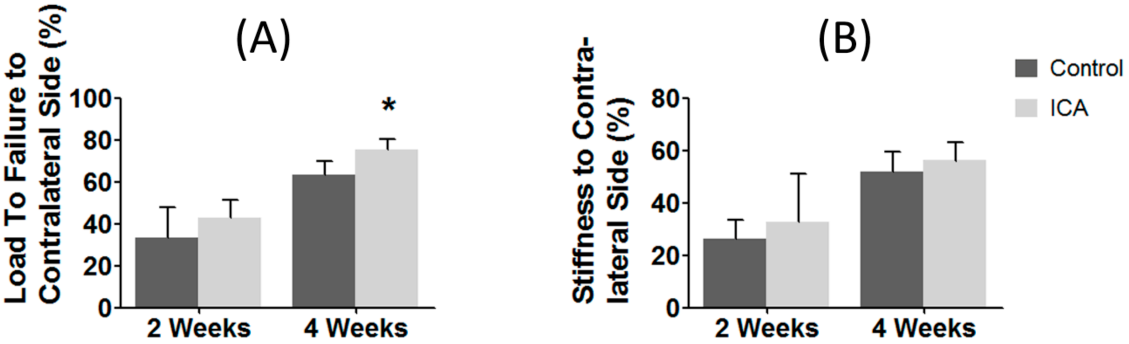

Figure S1. Compared to the healthy contralateral side, all groups had a significant lower load to failure and stiffness. No significant difference regarding the load to failure ratio (treated/contralateral) or stiffness ratio was found between control group and ICA treated group in 2 weeks after operation. However, the results of our study showed ICA treatment significantly improved the load to failure ratio in 4 weeks postoperatively. ICA also improved stiffness ratio in 4 weeks postoperatively, however, this improvement didn't reach statistical significance ($p > 0.05$). ICA: icariin. * $p < 0.05$. (**A**) load to failure ratio (treated/contralateral side); (**B**) stiffness ratio (treated/contralateral side).

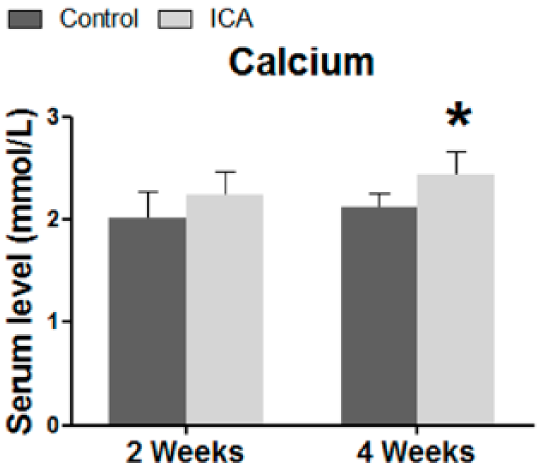

Figure S2. Serum chemistry. The results of ELISA showed an interesting trend towards higher serum calcium level in the ICA treated group. However, so far no similar results have been reported, the mechanism of this finding, remained unknown. * $p < 0.05$ vs. the control group.

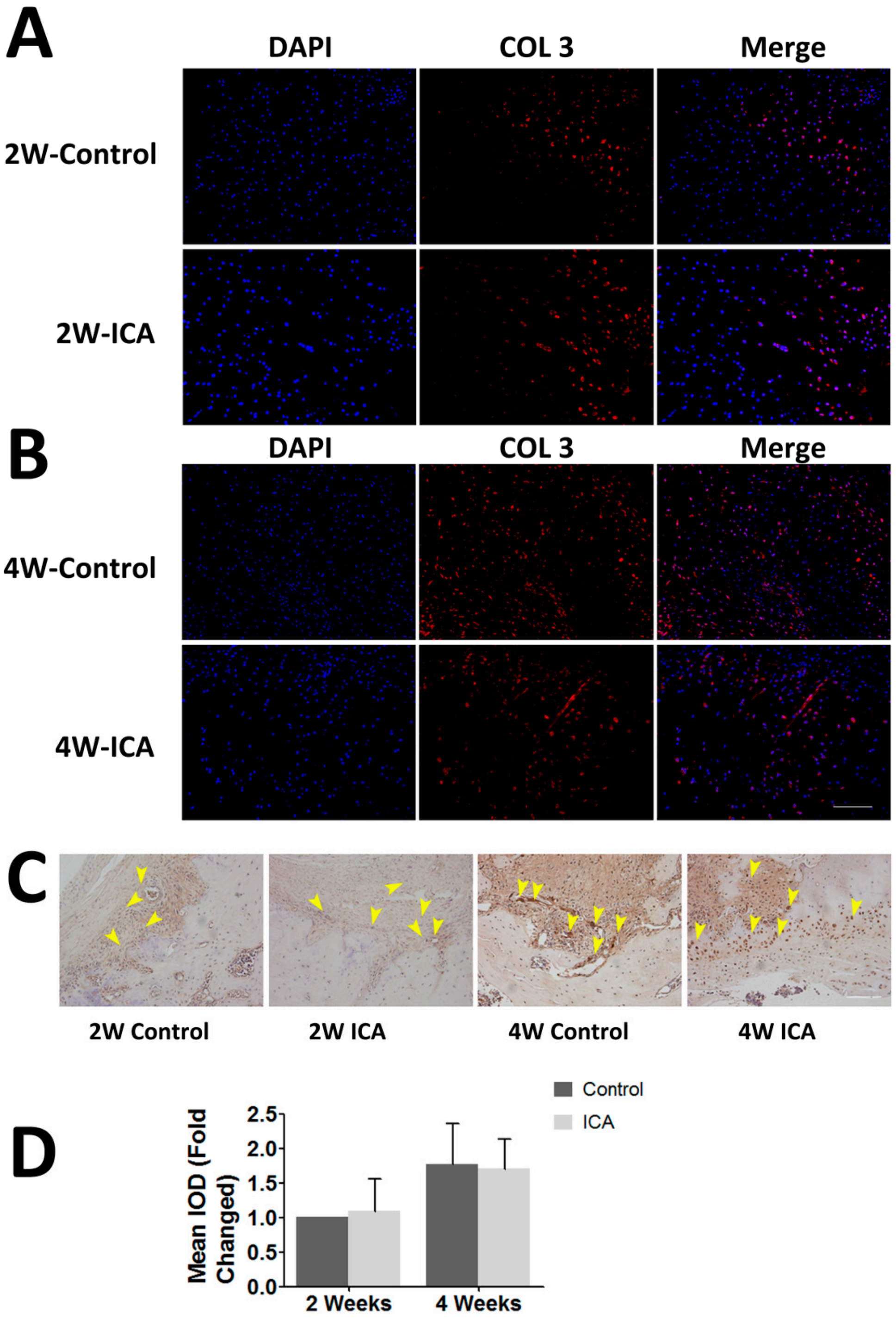

Figure S3. No significant difference regarding the results of immunohistochemical and immuno-fluorescence staining of Col3. (**A**) Immunofluorescence staining of Col3 in 2 weeks postoperatively; (**B**) Immuno-fluorescence staining of Col3 in 4 weeks postoperatively; (**C**) Immunohistochemical staining of Col3; and (**D**) Results of semi-quantitative analyses using Image J software (National Institutes of Health) showed that there was no significant difference regarding the Col3 protein expression between ICA treated group and control group. Arrow, Col3-positive cells. bar = 100 μm.
